# Daytime variation in hepatitis C virus replication kinetics following liver transplant

**DOI:** 10.12688/wellcomeopenres.14696.2

**Published:** 2018-09-20

**Authors:** Xiaodong Zhuang, Alvina G. Lai, Jane A. McKeating, Ian Rowe, Peter Balfe

**Affiliations:** 1 Nuffield Department of Medicine, University of Oxford, Oxford, Oxfordshire, OX3 7AZ, UK; 2 Institute for Data Analytics, University of Leeds, Leeds, Yorkshire, UK; 3 Institute for Immunology and Immunotherapy, University of Birmingham, Birmingham, West Midlands, B15 2TT, UK

**Keywords:** HCV, Circadian rhythm, liver transplant, allograft, viral rebound, time of day

## Abstract

**Background:** There is a growing interest in the role of circadian regulated pathways in disease pathogenesis.

**Methods:** In a cohort of hepatitis C virus (HCV) infected patients undergoing liver transplantation, we observed differences in early viral infection kinetics of the allograft that associated with the time of liver transplant.

**Results:** A higher frequency of subjects transplanted in the morning showed a rebound in viral RNA levels (n=4/6) during the first week post-surgery. In contrast, no viral rebound was observed in seven subjects transplanted in the afternoon. None of the other parameters previously reported to influence viral replication in the post-transplant setting, such as donor age, cold-ischemia time and length of surgery associated with viral rebound.

**Conclusions:** These observation highlights a role for circadian processes to regulate HCV infection of the liver and warrants further investigation.

## Introduction

The circadian clock is an evolutionarily conserved biological time-keeping system that synchronizes behavioural and physiological processes to a 24-hour cycle, including cell proliferation and metabolism ^1^. The circadian system is recognized to regulate host innate and adaptive immune responses to microbial pathogens to conserve energy utilization ^2– 8^. The circadian system comprises a central clock in the suprachiasmatic nucleus of the hypothalamus and secondary clocks in the peripheral organs. The liver is a highly circadian regulated organ with up to 20% of genes under clock control ^9^. Research over the past two decades has demonstrated that disrupting clock function associates with the development of liver diseases, including fatty liver disease, cirrhosis and hepatocellular carcinoma (HCC), highlighting a key role for the circadian system in regulating hepatic function ^9, 10^.

Viral infection of the liver is a global health problem with up to 71 million individuals infected with hepatitis C virus (HCV) that causes progressive liver disease and is one of the leading indications for liver transplantation ^11^. In almost every case HCV infects the newly transplanted organ or donor allograft, providing an unprecedented window to study the early stages of HCV infection. We had the opportunity to study the relationship between the time of liver transplantation and HCV replication dynamics in subjects enrolled in a clinical trial to assess the safety and efficacy of an entry inhibitor targeting scavenger receptor BI (SR-BI) ^12^. We noted differences in viral infection of the allograft in control subjects that associated with the time of liver transplant, suggesting a role for circadian processes to regulate HCV entry into the liver.

## Results

**Figure 1.**
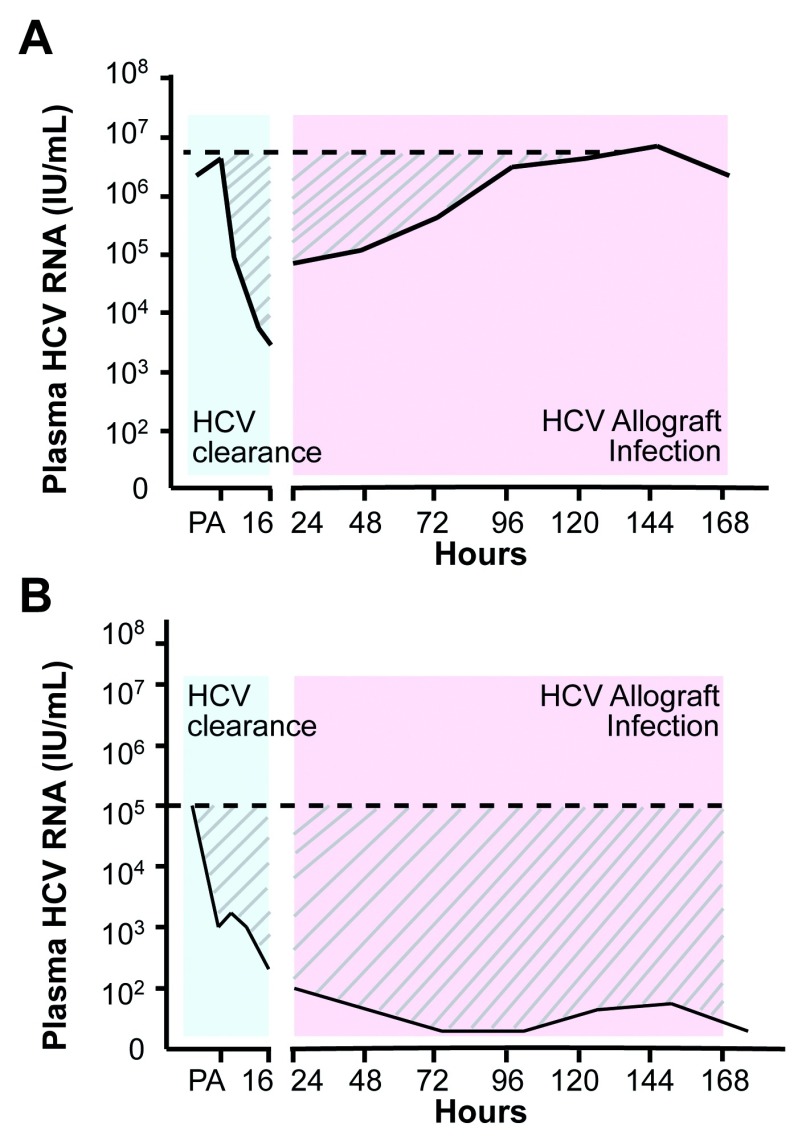
Schematic of hepatitis C virus (HCV) replication kinetics in the first week following liver transplant. Representative plasma HCV RNA levels are shown over the first week post-transplant. The declining values observed in the first 24 h following the post-anhepatic (PA) phase represent viral clearance (blue shading) from the periphery. Infection of the allograft results in a subsequent increase in viral RNA that can occur with either ‘rapid’ ( **A**) or ‘slow’ ( **B**) kinetics (pink shading). The horizontal dashed line represents the plasma viral load pre-transplant and allows us to quantify viral replication kinetics by measuring the area under the curve (AUC) (the hatched area), a patient showing rapid infection will result in a smaller AUC value compared to one with slower kinetics.

**Figure 2.**
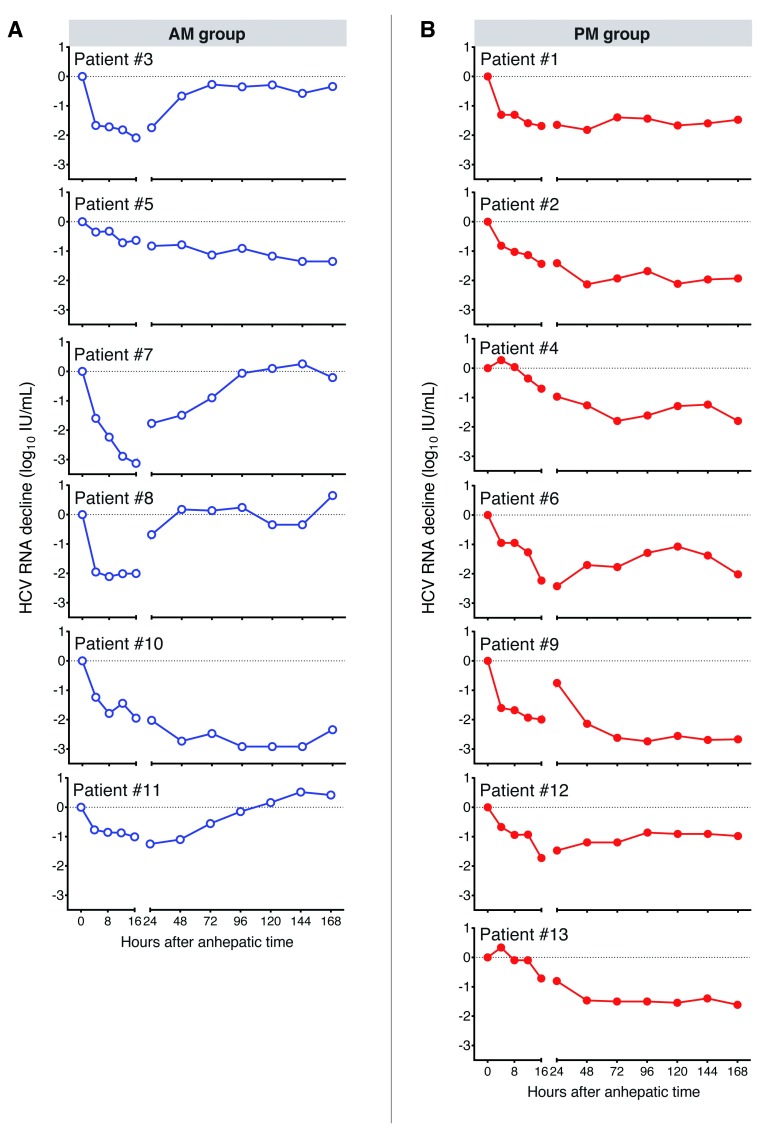
Hepatitis C virus (HCV) replication kinetics in control subjects in the AM or PM transplant groups. HCV RNA levels were quantified in subjects undergoing liver transplant in the AM ( **A**) or PM ( **B**) groups, with data expressed relative to the mean value of three samples collected after admission to hospital and before surgery. Samples were collected at 0, 4, 8, 12, 16 and 24 h post-transplant and daily thereafter for 7 days (168 h).

HCV infection of the newly transplanted graft is reported to show “rapid” or “slow” early phase replication kinetics ( Figure 1) ^13, 14^, however, the host pathways defining these profiles are not well understood. To investigate whether HCV replication kinetics is influenced by the time of transplant, patients in the untreated arm of the trial were grouped according to their time of surgery between the hours 6am–1pm (AM) (n=6) or 2pm–11pm (PM) (n=7). No patients were transplanted during the night (11pm–6am). Transplantation was required for liver failure (n=8) or HCC (n=5). Patients were infected with HCV genotype (Gt) 1 (7 patients) or Gt3 (4 patients), with single cases of Gt2 and Gt4. No significant differences in baseline median HCV RNA load were observed (5.4 log _10_ IU/ml) ( Table 1) ^15^. Additional clinical parameters previously reported to affect HCV replication or allograft survival, such as donor age (AM: median, 55 years; range 45–69 years; PM: median, 44 years; range, 29–64) ^16^, cold-ischemia time ^17^, duration and time of operation ^18^ were comparable in the AM or PM groups ( Table 1). In four of six patients (#3, 7, 8 and 11) in the AM group, a viral rebound toward pre-transplant levels was observed during the time of study ( Figure 2A). In contrast, none of the seven patients transplanted in the PM group showed a recovery of viral load to pre-transplant levels ( Figure 2B).

**Table 1.** Cohort data. Values shown for continuous variables are means (standard deviations).

Variable	AM (n=6)	PM (n=7)
Age, years	53 (11)	54 (7)
Sex		
Male	5	6
Female	1	1
Ethnicity		
Caucasian	4	5
Weight, kg	85 (11)	82 (16)
Indication for transplant		
Liver failure	3	5
HCC	3	2
MELD score	13 (4.5)	15 (3.7)
Initial HCV RNA, log _10_ IU/ml	5.0 (1.5)	5.7 (0.6)
Genotype		
Gt1	2	5
Non-Gt1	4 (2 Gt3, 1 Gt2, 1 Gt4)	2 (2 Gt 3)
Donor data		
Age, years	56 (8)	45 (11)
Cold Ischaemic time, mins	534 (64)	583 (142)
Duration of operation, h ¶	5.7 (2.0)	4.5 (0.8)

¶Duration estimated between the start of anhepatic phase and arrival on intensive care unit. One patient’s operation (AM) was 10 hours, all others were 4–6 hours. HCC, hepatocellular carcinoma; MELD, model for end-stage liver disease; HCV, hepatitis C virus.

**Figure 3.**
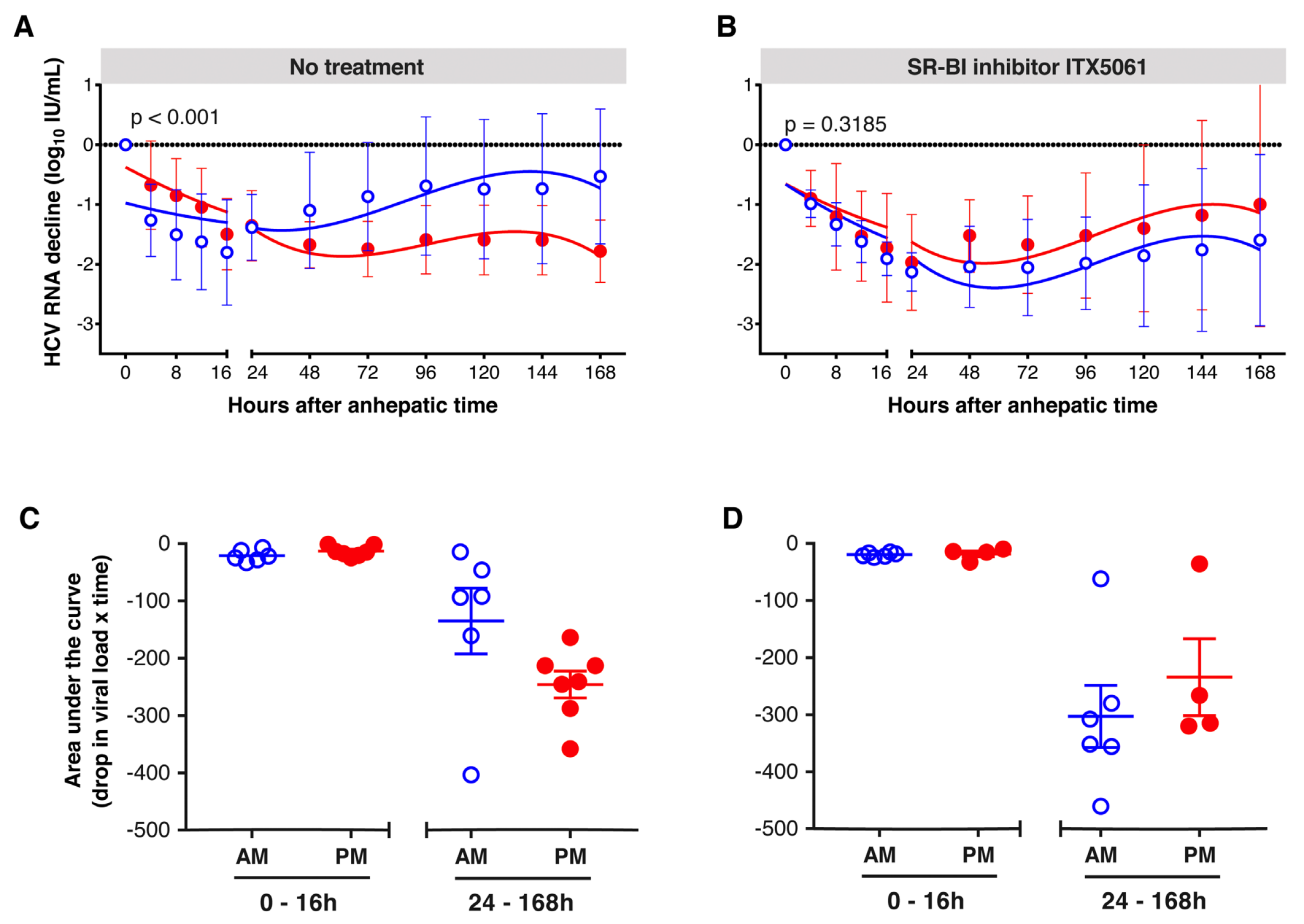
Effect of liver transplantation time on hepatitis C virus (HCV) clearance and early replication kinetics in control (n=13) and ITX5061 (n=10) treated subjects. The decline in HCV RNA levels in control ( **A**) and ITX5601 treated ( **B**) subjects in AM (blue) and PM (red) groups were averaged at each time point, plotted and lines of best fit calculated. Statistical comparison (F-test) showed a significant difference between viral replication in the control AM and PM groups (p<0.001). Patients were assessed for viral clearance (0–16 h) and infection kinetics (24–168 h) by determining the AUC over time in control ( **C**) and ITX5601 treated ( **D**) subjects where each symbol represents a patient. Groups were compared using a student’s unpaired t-test and no significant difference was observed.

Combining the replication kinetics from control subjects within AM or PM groups enabled us to apply lines of best fit and to conclude that the differences in replication kinetics were significant ( Figure 3A) (F-test, p<0.001). A similar analysis of patients receiving the SR-BI antagonist ITX5061 failed to show any significant difference in replication kinetics between the AM (n=4) or PM (n=6) groups ( Figure 3B), suggesting a role for circadian regulation of HCV entry.

We ^12^ and others ^13, 14^ have previously reported a rapid decrease in HCV RNA within the first 16 hours following surgery due to clearance of virus in the periphery by the reticular endothelial system of the new liver. To assess the influence of transplant time on viral clearance and allograft infection kinetics we calculated the area over the infection curve for control and treated subjects between 0–16 h and 24–168 h after transplantation ( Figure 3C, D). Time of transplant had minimal effect on viral clearance in all subjects ( Figure 3C, D). Control patients in the AM group showed higher rates of infection than those in the PM group, whereas this pattern was not apparent in the ITX5061-treated groups (AM, n=6; PM, n=4) and response to therapy was not time-dependent ( Figure 3C, D). Structuring the data in this manner did not reach significance and this most likely reflects the small number of data points analysed relative to the earlier regression analysis ( Figure 3A, B). In summary, these data support an association between HCV allograft infection rate and time of liver transplantation.

## Discussion

Our observation that the time of day of liver transplantation is associated with HCV allograft infection kinetics supports a role for circadian components to regulate host pathways important for HCV entry and replication. This observation has biological plausibility, since factors known to control both HCV entry and replication are reported to be under circadian control. For example, the tight junction proteins occludin and claudin-1, which define HCV entry into hepatocytes, have been reported to be circadian regulated in the colon ^19^, supporting a model where HCV entry into the liver may be circadian regulated. Similarly, the abundant liver-specific microRNA, miR-122, an essential host factor required for HCV RNA replication, is circadian regulated, and miR122 targeted genes showed clear circadian profiles ^20^, providing a further pathway for circadian control of HCV RNA replication.

Natural killer cells ^21^ and interferons ^22^ are major contributors to anti-viral responses, and are reported to be circadian regulated. In the context of liver transplantation, where recipients are immunosuppressed, the impact of recipient or allograft innate and adaptive immunity may be compromised, suggesting that differences in viral kinetics may reflect differences in hepatocellular permissivity to support HCV infection.

We recognize the limitations of this analysis, particularly with respect to the small number of patients studied. There are obvious ethical constraints in accessing donor liver tissue to assess its circadian status. However, to the best of our knowledge this is the first report highlighting a potential role for liver time-of-day regulated pathways to modulate HCV replication *in vivo* and this has clear translational potential for other hepatotropic infectious agents and the design of therapeutics.

## Methods

### Subjects

The data presented were obtained from subjects enrolled in an open-label phase 1b study to assess the effect of ITX5061 in patients undergoing liver transplantation at a single centre (Queen Elizabeth Hospital Birmingham, Birmingham, UK), described previously ^12^. All patients gave informed written consent and ethical approval was given by the UK National Research Ethics Service (reference 10/H0301/36) ^12^. The study was registered at clinicaltrials.gov ( NCT01292824). The study enrolled men and women between the ages of 18 and 65 years who were suitable for liver transplantation. Patients with HCV-associated end-stage liver disease or HCC were enrolled regardless of their infecting Gt or previous anti-viral treatment. Patients co-infected with HBV or human immunodeficiency virus were excluded, as were patients receiving a liver from a HCV-positive donor.

### Plasma collection and analysis

Plasma was collected at screening, before surgery, at the time of transplantation, and during a follow-up period of 90 days. HCV RNA levels were measured on admission to the hospital, immediately following the induction of anesthesia, at the time of portal vein clamping (the start of the anhepatic phase), immediately before perfusion of the allograft, and 1 hour later. Plasma samples were collected every 4 hours during the first post-transplant day, daily for the first week, weekly for the first month, and monthly thereafter up to 90 days. Plasma HCV RNA was measured using the COBAS TaqMan HCV test version 2.0 (Roche Diagnostics Ltd., Switzerland) in a laboratory accredited by the Health Protection Agency UK. Data were analysed using t-tests or F-tests in GraphPad Prism 7.0 software.

## Data availability

Raw data for the study, including demographic information for untreated transplant patients and hepatitis C virus RNA levels in untreated and ITX5061-treated groups, are available on OSF: http://dx.doi.org/10.17605/osf.io/kjnhr ^15^. Data are available under the terms of the Creative Commons Zero "No rights reserved" data waiver (CC0 1.0 Public domain dedication).
