# Supplementary material for: Daytime variation in hepatitis C virus replication kinetics following liver transplant
Source: Wellcome Open Res. 2018 Sep 20;3:96. Originally published 2018 Aug 7. [Version 2] doi: 10.12688/wellcomeopenres.14696.2 (PMC6107978; doi:10.12688/wellcomeopenres.14696.2)
Supplement: Peer review history

# Peer review: Referee response for version 2

**DOI:** 10.21956/wellcomeopenres.16122.r33952

**Published:** 2018-09-28

**Authors:** Koichi Watashi

**Affiliations:** 1 Department of Virology II, National Institute of Infectious Diseases, Tokyo, Japan

The authors appropriately responded to the reviewer's comments with opening the current available information.

I have read this submission. I believe that I have an appropriate level of expertise to confirm that it is of an acceptable scientific standard.

# Peer review: Referee response for version 1

**DOI:** 10.21956/wellcomeopenres.16003.r33655

**Published:** 2018-08-21

**Authors:** Arvind H. Patel

**Affiliations:** 1 MRC (Medical Research Council) - Centre for Virus Research, University of Glasgow, Glasgow, UK

This is an interesting study investigating the role of the circadian clock in hepatitis C virus (HCV) infection and replication. Using samples of HCV-infected liver transplant recipients enrolled in a clinical trial assessing the efficacy of a virus entry inhibitor ITX5061, the authors investigated replication kinetics of the virus and asked whether they are affected by the timing of the allograft. Previous studies have reported rapid or slow early phase virus replication in the new graft but the mechanisms remain unknown. Here the authors determined viral load at multiple time points up to 1 week in samples collected from untreated individuals who received the new liver in the morning (n=6, the AM group) or in the afternoon (n=7, PM). Remarkably, in 4 out 6 patients in the AM group viral rebound approaching pre-transplant levels was observed. In contrast, no increase in viral load was seen in any of the patients in the PM group. This difference was statistically significant allowing the authors to suggest that circadian processes play a role in regulating HCV infection. The authors also performed similar analysis in transplant recipients who were treated with the virus entry inhibitor ITX5061 and found no significant difference in the viral replication kinetics between the AM and PM groups allowing the authors to suggest circadian regulation of HCV entry. Notwithstanding the small sample size, the authors’ observations are very interesting and warrant further investigation to elucidate pathways and regulation of viral and host factors that modulate viral infection and replication in vivo.

I have read this submission. I believe that I have an appropriate level of expertise to confirm that it is of an acceptable scientific standard.

# Peer review: Referee response for version 1

**DOI:** 10.21956/wellcomeopenres.16003.r33652

**Published:** 2018-08-21

**Authors:** Graham R. Foster

**Affiliations:** 1 The Liver Unit, Blizard Institute, Queen Mary University of London, London, UK

This is an interesting short report examining the hypothesis that innate factors facilitating the early stages of the HCV replication cycle are under circadian control. The authors suggest that HCV entry and early replication is reduced in the afternoon. In support of this they show data from a detailed analysis of viral kinetics post liver transplantation and they compare a group undergoing surgery in the morning with those transplanted in the afternoon. The groups are reasonably well matched and obvious co-founders (genotype, duration of surgery) are accounted for. I presume that all donors were heart beating and that there were no gross differences between them but it would be helpful if the authors could confirm this and exclude the possibility that the observations are due to the use of ‘poor quality’ organs from non-heart beating donors in the operations performed in the afternoon. It would also be helpful to add in the viral genotypes to Figure 2 – there are some differences in the patterns within the groups and it would be helpful to see the figures annotated with viral genotype.

 The authors are appropriately guarded in their conclusions and make sensible suggestions regarding the underlying mechanisms. However, I would be interested in their thoughts on whether this finding should promote consideration of studies varying the timing of the administration of all oral antiviral agents – I wonder if drugs administered in the afternoon when viral spread is less active might be more effective and permit reduced treatment durations compared to medication taken in the morning.

 Minor comments:

 The reference to the total population infected with HCV is a little old and more recent estimates suggest that the prevalence is somewhat less than reported here.

I have read this submission. I believe that I have an appropriate level of expertise to confirm that it is of an acceptable scientific standard.

# Response

**Published:** 2018-09-04

**Authors:** Peter Balfe

**Affiliations:** University of Birmingham, UK

Referee : "it would be helpful to see the figures annotated with viral genotype."

 Response : Please see on line data where this data is given

 Referee : "I wonder if drugs administered in the afternoon when viral spread is less active might be more effective and permit reduced treatment durations compared to medication taken in the morning."

 Response : An interesting idea that could be relevant to anti-viral treatment for a range of human and animal pathogens. Our observations may inform future the design of pre-clinical studies assessing PK and drug efficacy. 

 Referee : "The reference to the total population infected with HCV is a little old and more recent estimates suggest that the prevalence is somewhat less than reported here.

 Response : We’ve updated this citation to the more recent WHO report from 2017, which suggest 71 million chronic infections exist ( http://www.who.int/entity/hepatitis/publications/global-hepatitis-report2017/en/index.html)

# Peer review: Referee response for version 1

**DOI:** 10.21956/wellcomeopenres.16003.r33653

**Published:** 2018-08-21

**Authors:** Koichi Watashi

**Affiliations:** 1 Department of Virology II, National Institute of Infectious Diseases, Tokyo, Japan

Zhuang and colleagues reported the difference in HCV recurrent dynamics that was concluded to depend on the time of transplantation in a small scale number of patients after liver transplantation: They showed that patients transplanted in the morning showed a high frequency of rapid HCV recurrence compared with those transplanted in the afternoon. No patients receiving transplantation in the afternoon showed a HCV rebound to the original level before transplantation.

 This paper shows a clinical evidence prospecting a surprising idea that HCV recurrence is reflected by circadian. Although it would be better to have more patient numbers and different hospitals, as the authors discussed, this paper will potentially trigger further analyses for larger scale study as well as the mechanistic study about the relationship between HCV infection and circadian. The specific comments to improve the paper are shown below.

 About the background of patients between two groups, it is reported that IL28B genotypes is associated with HCV recurrence after liver transplantation (Charlton et al. Hepatology 53: 317-324, 2011, and other papers). It would be nice to show the IL28B genotypes of donor/recipient of the two groups to neglect the possibility of the potential influence by different IL28B genotypes.

 The average duration of operation in the PM group (4.5 h) was shorter than that in the AM group (5.7 h). Showing an evidence or some citations suggesting that such difference will not have a significant impact on the clinical outcome is needed, especially for the present paper suggesting a surprising conclusion.

 Fig. 3 shows an interesting result that a SR-BI inhibitor cancelled the difference in HCV recurrent dynamics observed in non-treatment patients. Does it mean that SR-BI is regulated by circadian mechanism and the SR-BI inhibitor treatment reduces this influence on HCV infection? If it does, is there any information or previous papers suggesting that SR-BI is regulated by circadian mechanism? Or if it does not, discussion about the mechanism of the phenotype induced by the SR-BI inhibitor would be helpful.

 The authors described “supporting a role for circadian regulation of HCV entry” in line 5 of the right column on page 2, on which more information or citations are needed.

I have read this submission. I believe that I have an appropriate level of expertise to confirm that it is of an acceptable scientific standard, however I have significant reservations, as outlined above.

# Response

**Published:** 2018-09-04

**Authors:** Peter Balfe

**Affiliations:** University of Birmingham, UK

Referee : "It would be nice to show the IL28B genotypes of donor/recipient of the two groups to neglect the possibility of the potential influence by different IL28B genotypes."

 Response :  We thank the reviewer for this excellent suggestion, however, due to ethical issues it was not possible to genotype donor liver IL28B status. Since HCV early replication kinetics is most likely to be defined by the donor liver we decided to not genotype the transplant recipient.

 Referee : "​​​​​​​The average duration of operation in the PM group (4.5 h) was shorter than that in the AM group (5.7 h). Showing an evidence or some citations suggesting that such difference will not have a significant impact on the clinical outcome is needed, especially for the present paper suggesting a surprising conclusion."

 Response : The small cohort size means that a single observation can have a large impact. In this cohort there is one case (patient 1) where the operation took 10h (see on line data file), excluding this atypical data point resulted in mean operation times of 4.5h and 5h, respectively. 

 Referee : "​​​​​​​Fig. 3 shows an interesting result that a SR-BI inhibitor cancelled the difference in HCV recurrent dynamics observed in non-treatment patients. Does it mean that SR-BI is regulated by circadian mechanism and the SR-BI inhibitor treatment reduces this influence on HCV infection? If it does, is there any information or previous papers suggesting that SR-BI is regulated by circadian mechanism? Or if it does not, discussion about the mechanism of the phenotype induced by the SR-BI inhibitor would be helpful.

 Response : We previously reported that the SR-BI antagonist ITX5061 inhibited HCV rebound post liver transplant (Rowe et al. 2016) and hence it was not possible to discern any time-of-day effect in the treated arm of this trial.

 Referee : "​​​​​​​The authors described “supporting a role for circadian regulation of HCV entry” in line 5 of the right column on page 2, on which more information or citations are needed."

 Response : There is no additional information or citations showing a role for the circadian regulation of HCV entry factors. We have therefore revised this sentence to read ‘suggesting’ rather than ‘supporting’.

# Peer review: Referee response for version 1

**DOI:** 10.21956/wellcomeopenres.16003.r33651

**Published:** 2018-08-17

**Authors:** Salim I. Khakhoo

**Affiliations:** 1 Clinical and Experimental Sciences, Faculty of Medicine, Sir Henry Wellcome Laboratories, Southampton General Hospital, University of Southampton, Southampton, UK

The authors have performed a novel study investigating the role of the circadian clock in HCV infection.  They have studied 13 patients with chronic HCV infection undergoing liver transplantation either for liver failure or for hepatocellular carcinoma.  The study was performed on the back of a trial of an HCV entry inhibitor (ITX5061), which is designed to block reinfection of the liver allograft.  They have performed serial HCV viral load measurements in the plasma post transplantation to determine the HCV kinetics during the week after transplantation.  They stratify patients into those transplanted in the morning an those in the afternoon.  They find that individuals transplanted in the morning were more likely to have had a rebound in viral load.  This rebound begins 24-48 hours after the anhepatic phase in most of the individuals and in fig 3A appears to peak at around day 5.  The sample size is small but in the untreated individuals is significant.  This effect appears lost in the treated group.  Overall the study is well conducted and interesting

 Comments

1. It is not clear why chose the authors have chosen the specific intervals.  Is there a biological rationale, or was it for sample size convenience?
2. It is not clear to me which patients had received ITX5061 (this could be indicated in Figure 2).
3. The number of patients in each group should be indicated in Figure 3.
4. In figure 3 there are treated and untreated individuals.  There are 13 patients in Figure 3C are 13 and an additional 9 patients in Figure 3D.  These do not appear to be included in Table 1.  They should be included.
5. How ITX5061 was used is not clear to me.  Was it a single dose? Multiple doses? An Infusion? They need to clarify the statement “supporting a role for circadian regulation…” P2 para 1, as presumably it was given during the anhepatic phase for all patients.
6. How does the timing reflect doses of immunosuppression given?  Could this also have an effect on the post-transplant kinetic?

I have read this submission. I believe that I have an appropriate level of expertise to confirm that it is of an acceptable scientific standard.

# Response

**Published:** 2018-09-04

**Authors:** Peter Balfe

**Affiliations:** University of Birmingham, UK

Referee: "It is not clear why chose the authors have chosen the specific intervals.  Is there a biological rationale, or was it for sample size convenience? 

 Response : The intervals were selected to maximise sampling from the earliest times post transplant (4 hourly) and to monitor the treated patients throughout the time of ITX5061 administration (see Rowe at el 2016 for details).

 Referee list of detailed comments, with our reponses interleaved in *italics* :

1. It is not clear to me which patients had received ITX5061 (this could be indicated in Figure 2).
*None of the patients in Fig.2 received ITX5061. The treated patients are only shown in Fig.3B, this is now stated in the figure legend.*
2. The number of patients in each group should be indicated in Figure 3.
*We have edited the figure legend to include these data*
3. In figure 3 there are treated and untreated individuals.  There are 13 patients in Figure 3C are 13 and an additional 9 patients in Figure 3D.  These do not appear to be included in Table 1.  They should be included.
*Clinical information for the ITX5061 treated patients was previously published (Rowe 2016) and we cited this publication rather than duplicating data in current report.*
4. How ITX5061 was used is not clear to me.  Was it a single dose? Multiple doses? An Infusion? They need to clarify the statement “supporting a role for circadian regulation…” P2 para 1, as presumably it was given during the anhepatic phase for all patients. 
*ITX5061 administration details were provided in our earlier report (Rowe 2016) and we cited this publication rather than duplicating data in current report.*
5. How does the timing reflect doses of immunosuppression given?  Could this also have an effect on the post-transplant kinetic? 
*All patients arriving at the intensive care unit received standard immunosuppressive therapy of corticosteroids together with tacrolimus and azathioprine (details provided in Rowe 2016). Since our report studied HCV replication in the first week post-transplant when patients received the same immunosuppressive regimen we can exclude this parameter as a confounding influence.*

# Peer review: Referee response for version 1

**DOI:** 10.21956/wellcomeopenres.16003.r33654

**Published:** 2018-08-14

**Authors:** William L. Irving

**Affiliations:** 1 NIHR Nottingham Biomedical Research Centre, Nottingham University Hospitals NHS Trust, University of Nottingham, Nottingham, UK

This paper describes HCV viral replication kinetics in patients in the 7 day period following liver transplantation. To do this, the authors had access to a highly unique set of samples from patients undergoing transplantation in the control arm of a clinical trial of a potential HCV entry inhibitor. The key observation was made that in 4 of 6 patients whose transplant operation occurred between the hours of 6am to 1pm (the AM group), viral load rebounded to pre-transplant levels within the week of follow-up, compared to 0 of 7 patients whose operation took place between the hours of 2pm and 11pm (the PM group). The difference in viral replication kinetics of the 2 groups was statistically significant. For trial patients who received the trial entry inhibitor, there was no difference in replication kinetics. The time of transplant had minimal effect on the initial decline in viral load (clearance of virus from the periphery by the new allograft) in the first 16 hours post-transplantation, but rebound of viral replication, assessed as "the area over the infection curve") was greater in the AM group, although this did not achieve statistical significance, most likely due to the small numbers of patients in each group. In summary, the data are strongly suggestive of an association between HCV allograft infection rate and the time of day of transplantation, and the authors interpret these data to suggest a role for circadian processes to regulate HCV entry into the liver.

 The authors acknowledge 2 major limitations to this work – firstly, the small numbers of patients studied, and secondly, the absence of more direct data relating to the circadian status of the donor liver, for which there are obvious ethical constraints. Nevertheless, the observations made using this unusual set of samples are of interest in themselves, and raise further questions. If I have interpreted the data correctly, the implication is that whilst all of the implants appear to take up virus equally well, at least in the first 16 hours, it is only livers implanted in the AM period where the expression of factors underpinning either viral entry or those necessary for rapid viral replication (or both) just happen to be at an optimal level such that viral rebound becomes apparent soon after that initial 16 hour “mopping-up” period. I would be interested to hear the authors’ views on two issues related to this:

1. How important might be the time of day of removal of the donor liver? Is it a fair assumption that the circadian status of this liver will become fixed (almost literally frozen) as it is removed from neuronal and hormonal influences at the time of removal? Presumably data on time of removal will be available, although this is not mentioned in the manuscript. Was there also a correlation between viral replication kinetics in the recipient and time of day of removal of the donor organ?
2. A related question, to which the answer most probably will be pure speculation, is how long would it take for the donor liver to become synchronised to the circadian pattern of the recipient? The liver would be exposed to circulating humoral factors immediately, but there would be an absence of neuronal connections.

A couple of minor points for clarification:

1. The mean duration of the transplant operation was around 5 hours. So would it be true to say, for instance, that there were no operations that began at 11am and continued until 4pm ie straddling the time definitions for AM and PM? Or if that is not true, then into which category would such an operation be classified – AM according to the start time or PM according to the finish time?
2. The authors dismiss any possible confounding by the age of the donor liver – but the median age of the PM livers, at 44 years, was less than the entire range of the AM livers (45-69 years, 1 ^st^ para of the results). Liver age is a critical factor in both patients with chronic HCV infection (the older age at infection, the more likely disease progression) and the long-term prognosis of liver transplant recipients. Is it not conceivable that this might also be a factor in the PM group being better able to suppress HCV replication, at least in the early post-transplantation phase? Was the donor age in the 4 AM patients who rebounded any different from the donor age of the remaining patients?

I have read this submission. I believe that I have an appropriate level of expertise to confirm that it is of an acceptable scientific standard.

# Response

**Published:** 2018-09-04

**Authors:** Peter Balfe

**Affiliations:** University of Birmingham, UK

Referee : *"*How important might be the time of day of removal of the donor liver? Is it a fair assumption that the circadian status of this liver will become fixed (almost literally frozen) as it is removed from neuronal and hormonal influences at the time of removal? Presumably data on time of removal will be available, although this is not mentioned in the manuscript. Was there also a correlation between viral replication kinetics in the recipient and time of day of removal of the donor organ?"

 Response : This is an interesting question but as far as we are aware there are no previous reports on the circadian status of human liver explants. The cold ischaemic time of the donor livers are provided in the online material, from which time of explant can be calculated. There was no significant difference in the cold ischaemic time between the AM and PM groups and no association with post-transplant HCV replication kinetics.

 Referee : *"*A related question, to which the answer most probably will be pure speculation, is how long would it take for the donor liver to become synchronised to the circadian pattern of the recipient? The liver would be exposed to circulating humoral factors immediately, but there would be an absence of neuronal connections."

 Response : A really interesting question to which there is no authorative answer. PHH explanted from per2-luc transgenic mice can be synchronized in tissue culture and their circadian rhythm lasts around one week (Geunthner et al, PLoS One 2014 https://doi.org/10.1371/journal.pone.0087573). Furthermore their circadian rhythm can be re-synchronized by changing the culture media. This suggests that the circadian rhythm of liver explants is highly regulated by environmental factors such as hormones. From this one can speculate that the donor liver will rapidly adapt to the recipients’ rhythm post-transplantation.

 A related question, to which the answer most probably will be pure speculation, is how

 Referee : "The mean duration of the transplant operation was around 5 hours. So would it be true to say, for instance, that there were no operations that began at 11am and continued until 4pm ie straddling the time definitions for AM and PM? Or if that is not true, then into which category would such an operation be classified – AM according to the start time or PM according to the finish time?"

 Response : The length of all operations are listed in the online data, and with the exception of patient 1 in the PM group (10h), took 4 – 6h to complete. Many AM group patients did not arrive on the ICU until after 14:00. The end of the operation was defined as time of arrival/check in on the post-operative care ward, a significantly less precise parameter than the moment when anhepatic phase began. We therefore selected the start of the anhepatic phase as our time point to stratify the AM or PM groups. A second parameter to consider is the time of liver reperfusion, which occurred within 90 minutes of the anhepatic start time in the majority of cases. Indeed, if one stratifies patients by this criterion, the same groupings are obtained.

 Referee : "The authors dismiss any possible confounding by the age of the donor liver – but the median age of the PM livers, at 44 years, was less than the entire range of the AM livers (45-69 years, 1 ^st^ para of the results). Liver age is a critical factor in both patients with chronic HCV infection (the older age at infection, the more likely disease progression) and the long-term prognosis of liver transplant recipients. Is it not conceivable that this might also be a factor in the PM group being better able to suppress HCV replication, at least in the early post-transplantation phase? Was the donor age in the 4 AM patients who rebounded any different from the donor age of the remaining patients?

 Response : The age of all donors is provided in the online data. The four rebound cases received livers from donors aged 45, 56, 54 and 56 (average 53). One of the donors for the AM group was unusually old (69) and one of the PM donors unusually young (29), if these two cases are excluded then average age for the groups are 53 and 48 years,  respectively, supporting our conclusion that donor liver age is unlikely to explain the different viral replication kinetics in these two groups. However, as stated earlier greater patient numbers would strengthen our conclusions.
